# Association between Oxidative Stress with Psychological and Biochemical Variables in a Sample of Healthy Mexican People: A Cross-Sectional Study

**DOI:** 10.3390/antiox13010110

**Published:** 2024-01-17

**Authors:** Ana Míriam Saldaña-Cruz, Sergio Gabriel Gallardo-Moya, Liliana Campos-Medina, Aniel Jessica Leticia Brambila-Tapia

**Affiliations:** 1 Departamento de Fisiología, Centro Universitario de Ciencias de la Salud (CUCS), Universidad de Guadalajara, Guadalajara 44340, Mexico; ana.saldanac@academicos.udg.mx; 2 Doctorado en Farmacología, Centro Universitario de Ciencias de la Salud (CUCS), Universidad de Guadalajara, Guadalajara 44340, Mexico; sergio.gallardo@alumnos.udg.mx; 3 Departamento de Biología Molecular y Genómica, Centro Universitario de Ciencias de la Salud (CUCS), Universidad de Guadalajara, Guadalajara 44340, Mexico; liliana.campos5589@alumnos.udg.mx; 4 Departamento de Psicología Básica, Centro Universitario de Ciencias de la Salud (CUCS), Universidad de Guadalajara, Guadalajara 44340, Mexico

**Keywords:** oxidative stress, 8-OHdG, psychological variables, biochemical variables, depression

## Abstract

Oxidative stress (OS) has been linked to cell damage and chronic disease development; however, the study of psychological factors related with OS has been limited, as has its relationship with biochemical and personal variables. Therefore, the aim of this study was to evaluate the association between a wide variety of personal, psychological, and biochemical factors with OS in a sample of healthy Mexican people. A total of 134 participants, from which 70 (52%) were women, without known chronic conditions were included in the study, and the molecule 8-hydroxy-2′-deoxyguanosine (8-OHdG) was also measured as a marker of OS. We observed in the multivariate analysis of the whole sample that depressive symptoms (measured with CES-D scale) were the only psychological variable significantly associated (positively) with 8-OHdG. In addition, the following sociodemographic variables were associated with 8-OHdG: age, schooling (positively correlated), and the frequency of vitamins/antioxidant consumption (negatively correlated). The biochemical variables of erythrocytes in urine and amylase were positively correlated with 8-OHdG, while glucose was negatively correlated with it. Additional biochemical variables were associated in the multivariate analysis of each sex, including the positive correlation of LDL-cholesterol, LDH enzyme, lymphocytes, and the negative correlation of phosphorus and eosinophils in women’s samples, as well as the positive correlation of potassium, uric acid, and leucocytes in urine and the negative correlation of erythrocytes and lipase in the men’s samples. In conclusion, depression was the only psychological variable positively correlated with 8-OHdG after adjusting for confounders, and new associations with biochemical variables were found with some differences between sexes.

## 1. Introduction

Oxidative stress refers to the biological damage produced by free radicals [1]; however, it is considered a normal process in aerobic metabolism. It has been shown that reactive oxygen species (ROS) perform physiological roles in cellular signaling and in the defense against pathogens but, when they are present in excess, they can cause damage to macromolecules including lipids, DNA, and proteins, and can ultimately lead to cell death [1]. These ROS and reactive oxygen species of nitrogen and oxygen (RONS) are produced by several endogenous and exogenous processes, and their negative effects are neutralized by antioxidant defenses, which are also endogenous and exogenous [2]. It has been shown that oxidative stress is involved in several age-related conditions, including cardiovascular diseases, chronic kidney disease, neurodegenerative diseases, and cancer [2].

One of the main markers of oxidative stress in research studies is 8-hydroxy-2′-deoxyguanosine (8-OHdG), which is an oxidized derivative of deoxyguanosine and is the most abundant and investigated DNA lesion caused by oxidative stress, being the most precise and most often used biological marker of endogenous oxidative damage to DNA. Furthermore, 8-OHdG has been studied in relation to different chronic pathologies and environmental exposures in both sick and healthy populations [3]. This molecule has mutagenic properties and has been linked to cancer development, among other pathologies [3,4]. Although this marker has been studied in a variety of conditions and factors, its association with psychological variables has been limited. In this sense, a meta-analysis, which includes studies relating to depression, measured as a major depressive disorder or high depressive symptoms with the oxidative stress marker 8-OHdG, showed a positive association between depression and 8-OHdG [4]. Additional to depression, this marker has been associated with job stress models in the Japanese population [5]. However, the relation of 8-OHdG or additional oxidative stress markers with different psychological variables has not been performed to the best of our knowledge.

On the other hand, oxidative reactions have been associated with many biochemical variables and alterations, including anemia [6], extracellular hemoglobin [7], hyperlipidemia [8], and hyperuricemia [9]. However, a study evaluating the association between oxidative stress and a wide range of general biochemical variables, including serum electrolytes, in a relatively healthy population has not yet been performed, and many of these biochemical variables may have a positive or negative relationship with oxidative stress. In addition, although the relationship between oxidative stress and inflammation has been proposed, with inflammation being a cause of oxidative stress [10], we are unaware of any studies that evaluate the association between oxidative stress and inflammatory markers.

Therefore, the objectives of this study are (1) to determine the association of a wide variety of positive and negative psychological variables with 8-OHdG in a bivariate analysis and, after adjusting by sociodemographic and biochemical potential confounders, in a multivariate analysis, utilizing a sample of relatively healthy people; and (2) to determine the correlation between the inflammatory marker high-sensitivity C reactive protein (hs-CRP) with the oxidative stress marker 8-OHdG in a sample of relatively healthy people.

Based on these objectives, the hypotheses of this study are (1) that negative psychological variables (including depression, anxiety, and negative emotions) are positively correlated with the marker 8-OHdG, and positive psychological variables (including psychological wellbeing and emotional intelligence, among others) are negatively correlated with 8-OHdG, after controlling for personal, socio-demographic, and biochemical variables; (2) there is a positive correlation between the markers 8-OHdG and hs-CRP; and (3) there is a different correlation of biochemical variables with 8-OHdG in each sex.

## 2. Subjects and Methods

### 2.1. Ethical Considerations

The study was conducted according to the guidelines of the Declaration of Helsinki and was approved by the ethical committee of the Health Sciences University Center, with the registration number 19–21 (approval date 14 October 2019). All the participants signed an informed consent form. All personal and health-related data of participants were handled with strict confidentiality, being used only for research purposes.

### 2.2. Subjects

The inclusion criteria of the study were: (a) subjects older than 18 years old; (b) healthy subjects, this means subjects without chronic or acute diseases in the present moment known by the subject (auto-reported), and who were not taking any medication, although they could have presented symptoms of acute or even chronic diseases in the past, which was also measured; (c) who were not consuming illegal drugs (including marijuana); (d) who were not consuming hormonal products to increase muscular mass, (e) who were not pregnant; (f) who were not genetically related with another participant of the study (i.e., brothers, cousins); and (g) who preferably did not smoke. The elimination criterion was: (a) the absence of the measurement of any variable.

#### 2.2.1. Study Design

Cross-sectional study, by reason of no intervention of follow up measurements were performed.

#### 2.2.2. Sample Size

The sample size was calculated with the formula for bivariate correlations [11], which was estimated to detect a statistical confidence of 95% and a statistical power of 80%, for a minimum of correlation value of 0.2, which means a very low correlation. This formula yielded a total of 67 subjects. 

However, the minimum sample size intended was 80 individuals per sex. In this sense, we used this formula in order to detect very low bivariate correlations as significant, and we did not adjust for a specific number of independent variables because no formulas for multivariate analyses was found. However, with this formula we expected to detect clinically relevant correlations in the bivariate and multivariate analyses.

When multivariate analyses are carried out, it is desirable to use a large sample size in order to avoid a false result, with a minimum of 10 participants per variable; nevertheless, when this is not reachable, multivariate analyses can also be performed in order to diminish the confusion bias produced by the influence of multiple variables on a dependent variable, being cautious with the results’ interpretation.

#### 2.2.3. Procedures

The study was performed from July to November of 2022. The invitation was conducted by an announcement distributed via social networks (WhatsApp, Facebook) and personally to university students. All the potential participants met the inclusion criteria (by auto-report), and if they accepted the invitation to participate, were gathered (in groups from 11 to 20 participants) in the facilities of the University of Guadalajara. There, they signed an informed consent and filled out an electronic questionnaire that included personal and psychological variables. After filling out the electronic questionnaire, the anthropometric variables of body mass index (BMI) and waist-to-hip ratio (WHR) were obtained.

The blood and urine samples (to perform the laboratory tests) were obtained by qualified personnel (three biochemicals) who worked for a certified laboratory; they also obtained an additional blood sample in a dry tube for each participant, and this blood was destined for the extraction of the serum and measurement of the marker 8-OHdG in the university facilities.

After obtaining the samples, these were transported to a certified biochemical laboratory, where the biochemical analyses were performed by trained personnel.

### 2.3. Personal Variables

The following personal variables were obtained: sex; age; schooling; whether they had a job; having children; whether they had a romantic partner; socioeconomic level; daily free hours; daily hours of physical activity; monthly extra money, measured with 5 categories which ranged from nothing to more than 180 dollars; the frequency of alcohol and smoking consumption, measured with 5 categories from never to four or more times in the week. Sleep satisfaction was measured with the first item of the OVIEDO sleep questionnaire, and the answers options ranged from 1 (very unsatisfied) to 7 (very satisfied); sleep quality was measured with the second item (which in turn consists of 5 items) of the OVIEDO sleep questionnaire, with an answer range from 1 to 5 (low quality to high quality) [12]; and the quality of food intake was measured with the Mini-Ecca scale, which ranged from 1 (very low quality) to 12 (very high quality) [13]. Two additional questions of eating behavior were included: (a) the frequency of food consumption outside the home, and (b) the frequency of food consumption in excess, where both questions were measured with 7 answer options, from 1: less than once in a month to 7: all the days; these questions were obtained from the eating behavioral questionnaire [14]. Although an inclusion criterion was the absence of acute or chronic diseases in the present moment, we measured the presence of the 21 following diseases in the last 6 months: diabetes, hypertension, overweight, cancer, respiratory infections, gastrointestinal infections, allergies/asthma, gastritis/gastric ulcer, colitis/irritable colon, rheumatic diseases (rheumatoid arthritis, systemic lupus erythematosus, etc.), thyroid diseases, migraine, skin diseases (acne/neurodermatitis, etc.), sinusitis, kidney/bladder problems, anorexia/bulimia, heart attack/angina pectoris, cerebral stroke, high cholesterol levels, anxiety and depression problems that require medication) and any additional disease. Finally, the use of antioxidants/vitamins was recorded as a yes/no question, and the frequency of these supplements’ consumption was also recorded with 5 ordinal categories: from never to daily.

### 2.4. Measurement of Anthropometric Variables

After the filling out of the questionnaire, the height and weight were obtained by trained personnel with a Tanita brand scale (model bc-533) and a measuring tape attached to the wall, to calculate the BMI. The hip and waist circumferences were also obtained by trained personnel by using a measuring tape, and these measurements were used to calculate the WHR.

### 2.5. Psychological Variables

The following psychological variables were measured: depressive symptoms, with the 10-item CES-D scale, ranging from 1 (no days) to 4 (all the days) [15,16]; anxiety symptoms, with the generalized anxiety disorder test (GAD-7), ranging from 0 (never) to 3 (almost all the days) [17]; the presence of positive and negative emotions with the positivity-self scale (PSS), ranging from 1 (never) to 5 (almost always) [18]; the 6 subscales of the shortened version of the psychological well-being (PWB) scale (including: self-acceptance, environmental mastery, autonomy, personal growth, purpose in life, and positive relations with others), in which this scale ranged from 1 (totally disagree) to 6 (totally agree); [19]; optimism was measured with the life orientation test (LOT-R), from 1 (totally disagree) to 5 (totally agree) [20]; additionally, we measured 5–6 items of 4 subscales of the trait emotional intelligence questionnaire (TEIQUE): self-motivation (5 items), emotion perception (5 items), assertiveness (6 items), and emotion regulation (6 items), which ranged from 1 (totally disagree) to 7 (totally agree) [21], and these items are described in the Supplementary File S1. All the psychological instruments showed a Cronbach alpha value > 0.60, which indicates an acceptable reliability.

### 2.6. Biochemical Variables Measurement

Analyses of biochemical variables were performed in a private and certificated laboratory (with EMA and joint commission international certifications). Blood samples were obtained from all participants in order to quantify the following: (1) complete blood count test (including leucocytes and their subpopulations, erythrocytes, and hematocrit), (2) complete lipid profile test (total cholesterol, high-density lipoprotein (HDL), low-density lipoprotein (LDL), and triglycerides), (3) liver function tests (gamma glutamyl transferase (GGT), aspartate aminotransferase (AST), alanine aminotransferase (ALT), alkaline phosphatase (ALP), lactate dehydrogenase (LDH) enzymes and direct and indirect bilirubin, albumin, and total proteins), (4) blood chemistry (including creatinine, glucose urea, blood urea nitrogen (BUN), and uric acid), (5) serum electrolytes (calcium, phosphorus, magnesium, iron, sodium, potassium, and chloride), (6) pancreatic enzymes (amylase and lipase), and (7) a general urine test, including leukocyte esterase, erythrocytes, and leukocytes in urine. The high-sensitivity C reactive protein (hs-CRP) was measured with the ELISA technique, and all of the values out of range detected in the laboratory tests were double checked (performed twice) in order to verify them.

### 2.7. Serum Quantification of 8-OHdG

A serum sample of all participants was obtained by centrifugation and stored at −70 degrees Celsius until usage. In order to quantify the marker 8-OHdG, we used the ELISA technique (Mybiosource, Inc., San Diego, CA, USA), following the manufacturer’s instructions. The sensitivity of this assay is 0.938 ng/mL, the range of detection is 1.563–100 ng/mL, and the intra-assay precision is <8%.

### 2.8. Statistical Analysis

In order to perform the description of continuous variables, we used the mean and standard deviations when the distribution was parametric and the median and ranges when it was non-parametric. In order to compare sociodemographic variables between sexes we used the chi-squared test for qualitative variables; this test was used because it is indicated to perform comparisons between qualitative or categorical variables between 2 study groups. We used Student’s t-test or Mann–Whitney U test for quantitative variables (depending if the distribution was parametric or non-parametric); these tests were selected because they are the indicated ones to perform comparisons of continuous variables between 2 study groups, in this case males and females. In order to correlate quantitative variables with 8-OHdG, we used the Pearson and Spearman correlation tests, which are indicated for bivariate correlations between continuous variables, depending on the parametric or non-parametric distribution of the data. In order to determine the independent variables most correlated with oxidative stress, a multiple linear regression analysis (using the stepwise method) with 8-OHdG as the dependent variable was performed for all the samples and segmented by sex. This analysis was selected because the dependent variable (8-OHdG) was continuous and normally distributed and the independent variables were continuous, dichotomic, and ordinal, and the stepwise method was selected in order to obtain a model with all of the included variables being significant. This analysis permitted us to control the confounding effect of independent variables on the dependent variable, adjusting the significances obtained by all of the variables included.

Finally, the Cronbach’s alpha test was obtained for all of the psychological instruments (including the subscales) in order to obtain the reliability of each scale and subscale applied. All analyses were performed with the software SPSS v.25, and a *p* value < 0.05 was considered as significant.

## 3. Results

A total of 134 participants were included, from which 70 (52.2%) were women. The mean ± SD of the age of the whole sample was 26.04 ± 9.79 years old. All of the psychological and behavioral instruments employed had a Cronbach’s alpha test above 0.6.

The descriptive data of the sociodemographic, psychological, and behavioral variables are described in Table 1, where we can observe that both sexes were similar in most variables with the exception of age, the women’s sample being slightly older than the men’s (3 years on average). Likewise, the women’s sample had significantly more participants with children than the men’s sample, and the women’s sample had significantly more participants at the university schooling level than the men’s sample (Table 1). In addition, women had a higher quality of food intake than men and a lower frequency of food consumption outside the home than men. With respect to psychological variables, women had higher anxiety levels and more negative emotions than men, as well as less autonomy and assertiveness than men (Table 1). Regarding the consumption of vitamins and antioxidants, we observed that women had a higher consumption of these supplements than men, with a borderline *p* value (*p* = 0.052).

The levels of DNA oxidation measured by 8-OHdG in each sex are shown in Table 2, and no differences in these levels were observed between sexes (*p* = 0.357).

With regard to the main diseases experienced by the participants in the last 6 months, the diseases with a higher frequency in the women’s sample were respiratory infections (including COVID) (31.4%), colitis/irritable colon (27.1%), acne (27.1%), gastritis/gastric ulcer (21.4%) allergies (21.4%), gastrointestinal infections (18.6%), and migraine (12.9%); while in the men’s sample, the most frequent diseases reported were acne (31.3%), allergies (25.0%), gastrointestinal infections (15.7%), overweight (15.7%), and respiratory infections (14.1%). Although the percentages of each disease were different between sexes, we did not detect significant differences in the frequency of these percentages (*p* = 0.148), nor in the number of diseases presented between sexes (*p* = 0.145, Table 1).

The descriptive data of all biochemical variables included in the study are presented in Table 2, along with the normal reference values and the percentage of individuals out of the reference values in each sex. It is important to mention that the laboratory reference ranges are according to the desirable values and not the normal values found in the general population. Therefore, we included values out of range in the analyses by considering that these variations are expected in relatively healthy population and are useful in the searched associations. When a value out of range was detected in the laboratory, this was double checked to verify it.

### 3.1. Bivariate Correlations

In Table 3, we show the significant bivariate correlations of the whole sample and the samples segmented by sex. We observe that no psychological variables were associated with 8-OHdG. However, positive correlations with this marker were found in the whole sample for the sociodemographic variables of age, schooling, with children, and with job, and for the biochemical variables of total cholesterol, LDL-cholesterol, erythrocytes in urine, and potassium serum levels; while a low but significant correlation was found between phosphorus and 8-OHdG (Table 3). Additionally, men showed a significant low negative correlation between the sum of diseases and 8-OHdG, while women presented a low negative correlation between the use of antioxidant supplements and their frequency of consumption with 8-OHdG. Finally, no significant correlation was found between hs-CRP and 8-OHdG, either globally (*r* = −0.003) or separated by sex (men, *r* = −0.046, women, *r* = 0.018) (Table 3).

### 3.2. Multivariate Regression Analysis

In the multivariate regression analysis in the global sample, we found that age was the most positively associated variable with 8-OHdG, followed by schooling, erythrocytes in urine, depressive symptoms, and pancreatic amylase. The negatively associated variables were glucose and the frequency of antioxidant consumption (Table 4).

In the women’s sample, the most positively associated variables were erythrocytes in urine, LDL-cholesterol, sum of diseases, lymphocytes, and LDH; while the negatively associated variables were phosphorus in serum, frequency of antioxidants consumption, fasting glucose, and eosinophils (Table 5).

In men’s sample, the multivariate regression analysis showed the following positively correlated variables: erythrocytes in urine, schooling, age, potassium serum levels, uric acid, monthly extra money, and leukocytes in urine, while the negatively correlated variables were fasting glucose and the number of erythrocytes (Table 6).

## 4. Discussion

We observed that oxidative stress was associated with different personal, biochemical, and psychological variables in both the bivariate analysis and the multivariate regression analysis. With respect to the psychological variables, we detected a significant association with depressive symptoms in the multivariate analysis for the whole sample (Table 4); this finding coincides with the previous meta-analysis performed in different populations, where depression was significantly and positively associated with oxidative stress markers (including 8-OHdG and F-2 isoprostanes) [4]. However, all the studies included in the meta-analysis corresponded to case–control designs, where case groups were differenced either by high depressive symptoms, by the presence of bipolar disorder or major depressive disorder, but did not perform the analyses adjusted by a wide number of personal and biochemical variables as was performed in the present study. Therefore, our results corroborate the relationship between depressive symptoms with oxidative stress (measured with 8-OHdG), in a cross-sectional design after adjusting by many possible confounders. This relationship has been explained by the effect of chronic stress, leading to hyperactivity of the hypothalamic–pituitary–adrenal axis (HPAA) and an increase in the mitochondrial allostatic load which decreases energy production and elevates the generation of ROS and RONS, which ultimately predispose to depression and other chronic diseases [22]. These findings also coincide with a report showing a positive association between psychological stress and urinary 8-OHdg in a large sample of Japanese individuals, in which, although an association between depressive symptoms and urinary 8-OHdg was not reported [23], these observations are aligned with the psychological pathway involving the hyperactivity of the HPAA triggered by chronic stress, resulting in an increase in ROS and RONS. In addition, it has been shown that mitochondrial dysfunction, a product of chronic stress, in turn, affects the HPAA, modifying the stress response, catecholamine levels, and the IL-6 proinflammatory cytokine secretion, all of these being well-known mechanisms leading to disease, including depression [24].

In addition, although oxidative stress and inflammation are two linked pathways [10,24], we did not find a correlation between 8-OHdG and hs-CRP, either globally or separated by sex; this unexpected finding could be explained by the possibility that both variables are produced by similar mechanisms but with differences in time, duration, and also being modified by different conditions. However, the association between lymphocytes with 8-OHdG in the women’s sample (Table 5) and with leukocytes in urine in the men’s sample (Table 6) in the multivariate regression analyses supports a possible relationship between oxidative stress and inflammation. However, further studies exploring the relationship between oxidative DNA damage and inflammatory biomarkers, including proinflammatory cytokines and C reactive protein, should be performed in order to clearly determine the relationship between oxidative stress and the inflammatory pathways.

With respect to the associations between sociodemographic variables and 8-OHdG, the positive correlation found between age and 8-OHdG in both sexes coincides with the theory of oxidative stress and aging [2] which establishes an association between the accumulation of RONS with age and age-related conditions. The variable age was also significantly associated with 8-OHdG in the multivariate regression analysis of the whole sample and the men’s samples (Table 4 and Table 6), confirming this relationship. Additionally, the positive correlation between the sum of diseases in the last six months only in the women’s sample is also associated with the link between inflammation and oxidative stress previously mentioned [10], as well as with the relationship between oxidative stress and aging [2]. Nevertheless, the men’s sample did not show such an association and, in contrast, a negative low correlation was found in the bivariate analysis between 8-OHdG and the sum of diseases in the last six months (Table 3), which was not corroborated in the multivariate regression analysis. Therefore, this correlation can be due to the effect of other confounding variables and also to the different type of diseases presented in each sex, with women presenting more cases with migraine, gastritis, colitis, and respiratory infections than men, diseases that are typically related with inflammatory processes and probably with oxidative stress. Additionally, women showed a higher number of diseases than men, although this difference did not reach statistical significance probably due to the sample size.

We also found that schooling was positively correlated with 8-OHdG in both sexes (being higher in men) and in the multivariate analyses of them, this correlation can be explained by the oxidizing effect of sedentary behaviors previously shown in men [25]; this may also be explained by considering that a higher schooling level could be related with more sedentarism, both during the study period and in the kind of jobs obtained by people with a higher level of schooling. However, this association in each sex should be further explored. Additionally, the variable monthly extra money positively correlated with 8-OHdG in men’s multivariate analysis, an association that should also be further explored; although, this finding could be related to a possible increase in stress that could be associated with obtaining a higher income.

Finally, the variable “use of vitamins/antioxidants supplements” and its frequency of consumption was negatively correlated with 8-OHdG only in the women’s sample in the bivariate correlations (Table 3), as well as in the whole sample and the women’s samples in the multivariate regression analysis (Table 4 and Table 5). This correlation is explained by the protective effect of antioxidant supplements in general, and the fact that this correlation was mainly observed in women can be related with the fact that women consumed more supplements than men, with a borderline *p* value (Table 1). However, further studies evaluating the consumption of specific antioxidants and/or its presence in blood performed by sex would specify this finding. However, these results coincide with previous reports showing that vitamins and antioxidant supplements are effective in preventing chronic diseases [26] and in treating acute ones [27]. Nevertheless, specific compounds are more useful for specific diseases and individuals; therefore, more studies evaluating the effect of each compound consumption, their concentrations, and their clinical effects in clinically healthy and non-healthy populations will clarify these relationships.

With respect to biochemical variables, we found that many biochemical variables were significantly correlated with 8-OHdG either in the bivariate or multivariate analyses. The positive correlation between erythrocytes in urine and 8-OHdG in the multivariate analyses of the whole sample and of each sex is interesting. Although we could not detect a previous report searching for such an association, we found a previous publication showing that extracellular hemoglobin produces oxidative reactions [7] leading to oxidative products related with inflammatory effects and oxidative stress. This suggests that erythrocytes in the urine, as a product of infections or menstruation, could increase oxidative stress in the general system, a hypothesis that should be corroborated. We also found a positive correlation between LDL-cholesterol and 8-OHdG, an association that coincides with a previous report showing an association between oxidative stress and atherosclerosis, which is, in turn, related with hyperlipemia [8]; we observed that this correlation was present in both sexes in the bivariate analysis but only in the women’s sample in the multivariate analysis (Table 5) which suggests that this relationship is more pronounced in women and could be related with LDL oxidation occurring in high levels in women. In addition, the negative correlation between glucose and 8-OHdG, in the women’s sample in the bivariate analysis and in the three multivariate analyses (Table 4, Table 5 and Table 6), could be related with the fatty acid oxidation in low-glucose-level conditions [28,29], which could increase oxidative stress. It is important to consider that most subjects analyzed in this study were normoglycemic, with only three individuals with glucose levels out of range (Table 3); therefore, low glucose levels were expected to impact more clearly on fatty acid oxidation in this sample, which in turn would increase the oxidative stress.

In the multivariate analysis of the men’s sample, we observed a positive correlation between uric acid and 8-OHdG, a finding that coincides with a previous report associating hyperuricemia with oxidative stress [9]. This observation was detected only in the men’s sample probably because men had higher levels of uric acid than women (Table 2). In the men’s sample, we also detected a negative correlation between erythrocytes (in blood) and 8-OHdG in the multivariate regression analysis, a finding that coincides with previous reports showing an association between oxidative stress with eryptosis, erythrocyte damage, and anemia [6,30]. Interestingly, this association was found only in men, which needs to be further explored.

Finally, we found that phosphorus levels were negatively correlated with 8-OHdG in the bivariate analysis of the three samples (Table 3) and in the multivariate analysis of the women’s sample; this interesting finding was not found in previous reports, but could be related with the fact that phosphate-related molecules, such as ATP and DNA require phosphorus, so its negative correlation with oxidative stress (8-OHdG) could be related with an optimal mitochondrial function in ATP production and less RONS and ROS production in conditions of adequate levels of serum phosphate and/or with an improved system of DNA repair with adequate phosphate concentration, reducing the effects of free radicals in this molecule. However, these hypotheses should be further explored.

In addition, the positive correlation of potassium with 8-OHdG detected in the multivariate analysis of the men’s sample, along with other significant associations of the biochemical variables: LDH enzyme, pancreatic amylase, and lipase in the multivariate regression analyses, are new findings that require further investigation in order to clarify the relationship between these variables and oxidative stress. With respect to these observations, we did not find related references that could explain them, this considering that these specific relationships have not been investigated. However, the differences in the biochemical variables associated with 8-OHdG observed in each sex can be attributed to hormonal influences, unmeasured lifestyle factors, or sex-related genetic predispositions.

The main limitations of the study are the small sample size, which increases the random bias, and the cross-sectional design that does not permit us to establish causal associations because the possible causal factors do not precede the possible effects; therefore, in order to determine causality between any of the correlations found, longitudinal studies (either observational or experimental) should be performed. Another important limitation of the study is the use of a general scale to measure the quality of food intake, which does not permit the measurement of the intake of specific types of food, mainly fruits and vegetables; also, the lack of the employment of an instrument to measure physical activity did not permit us to measure the specific effect of this variable in a more reliable way. Finally, the lack of measurement of environmental exposures related with DNA damage (including solar and toxin exposure) could also have influenced the results observed. However, the inclusion of many personal, psychological, and biochemical variables diminished the confusion bias and increased the possibility of finding new associations with oxidative stress that can be further explored.

## 5. Conclusions

In conclusion, we found that, after controlling for several personal and biochemical variables, depressive symptoms are significantly correlated with oxidative stress, while no other negative or positive psychological variables were associated with it. In addition, many socio-demographic and biochemical variables were associated with oxidative stress, either in the global sample or in sex-specific samples, these variables included: age and schooling, which were positively associated with 8-OHdG; the consumption of vitamins/antioxidants, which was negatively associated with 8-OHdG; and the biochemical variables of erythrocytes in urine, LDL-cholesterol, potassium, uric acid, amylase, lymphocytes, and leucocytes in urine, which were positively correlated with 8-OHdG; and phosphorus, erythrocytes, eosinophils, and lipase, which were negatively correlated with 8-OHdG, in the multivariate analyses of either the global or specific sex samples. However, further studies with larger sample sizes and with experimental or observational longitudinal designs will corroborate or contradict these results.

## Figures and Tables

**Table 1:** Descriptive data of the sociodemographic and psychological variables of the included participants.

Variable	Women (*n* = 70)	Men (*n* = 64)	*p* Value
Age	27.49 ± 10.11	24.47 ± 9.26	0.006 *
With romantic partner, *n* (%)	32 (45.7)	32 (50)	0.729
With children, *n* (%)	18 (25.7)	5 (7.8)	0.006 *
With job, *n* (%)	37 (52.9)	28 (43.8)	0.305
Schooling, *n* (%)			0.032 *
Elementary school	1 (1.4)	0 (0.0)	
Secondary	3 (1.4)	2 (3.1)	
Preparatory	38 (54.3)	45 (70.3)	
University (Bachelor’s degree)	27 (38.6)	11 (17.2)	
Master’s degree	3 (4.3)	5 (7.8)	
Ph.D. degree	0 (0.0)	1 (1.6)	
Socioeconomic level, *n* (%)			0.196
Very low	0 (0.0)	1 (1.5)	
Low	11 (15.7)	13 (20.3)	
Average	58 (82.9)	46 (71.9)	
High	1 (1.4)	4 (6.3)	
Very high	0 (0.0)	0 (0.0)	
Monthly extra money, mean ± SD			0.106
Nothing	3 (4.3)	8 (12.5)	
Less than USD 60	26 (37.1)	12 (18.8)	
From USD 61 to USD 120	23 (32.9)	22 (34.4)	
From USD 121 to USD 180	6 (8.6)	8 (12.5)	
More than USD 180	12 (17.1)	14 (21.8)	
Smoking frequency, median (range)			0.193
Never	66 (94.3)	57 (89.1)	
Two to four times in the year	3 (4.3)	7 (10.9)	
Once a month or less	0 (0.0)	0 (0.0)	
Two to three times in a week	1 (1.4)	0 (0.0)	
Four or more times in a week	0 (0.0)	0 (0.0)	
Alcohol consumption frequency, median (range)			1.000
Never	12 (17.1)	12 (18.8)	
Two to four times in the year	23 (33.0)	21 (32.8)	
Once a month or less	29 (41.4)	26 (40.6)	
Two to three times in a week	5 (7.1)	5 (7.8)	
Four or more times in a week	1 (1.4)	0 (0.0)	
Daily free hours, mean ± SD	4.08 ± 2.13	4.97 ± 2.72	0.060
Daily physical activity hours, mean ± SD	1.24 ± 0.94	1.17 ± 0.84	0.807
Sleep satisfaction (OVIEDO scale), mean ± SD			0.515
Very unsatisfied	5 (7.1)	1 (1.6)	
Quite unsatisfied	6 (8.6)	7 (10.9)	
Unsatisfied	9 (12.9)	11 (17.2)	
Medium	25 (35.7)	20 (31.3)	
Satisfied	15 (21.4)	11 (17.2)	
Quite satisfied	7 (10.0)	7 (10.9)	
Very satisfied	3 (4.3)	7 (10.9)	
Sleep quality (OVIEDO scale), mean ± SD	3.61 ± 0.91	3.85 ± 0.87	0.125
Sum of diseases in the last 6 months, median (range)	2 (0–11)	1 (0–5)	0.145
Frequency of food consumption outside home, *n* (%)			0.042 *
Less than once a month	6 (8.6)	1 (1.6)	
Once a month	4 (5.7)	1 (1.6)	
Once every 15 days	10 (14.3)	10 (15.6)	
1–2 times a week	35 (50.0)	22 (34.3)	
3–4 times a week	6 (8.6)	12 (18.8)	
5–6 times a week	5 (7.1)	10 (15.6)	
Daily	4 (5.7)	8 (12.5)	
Frequency of food consumption in excess, *n* (%)			0.089
Less than once a month	11 (15.7)	3 (4.7)	
1 time at month	9 (12.9)	10 (15.6)	
1 time each 15 days	10 (14.3)	13 (20.3)	
1–2 times a week	28 (40.0)	23 (35.9)	
3–4 times a week	5 (7.1)	12 (18.8)	
5–6 times a week	5 (7.1)	1 (1.6)	
Daily	2 (2.9)	2 (3.1)	
Use of vitamins/antioxidant supplement, *n* (%)	20 (28.6)	11 (17.2)	0.152
Frequency of vitamins/antioxidant supplement consumption, *n* (%)			0.052
Never	51 (72.8)	53 (82.8)	
Less than once in the week	3 (4.3)	1 (1.6)	
1–2 times in the week	7 (10.0)	0 (0.0)	
3 to 4 times in the week	2 (2.9)	4 (6.2)	
Daily	7 (10.0)	6 (9.4)	
Quality of food intake (Mini-Ecca scale), mean ± SD	8.06 ± 2.48	7.28 ± 2.42	0.039 *
Psychological variables			
Anxiety (GAD-7), mean ± SD	1.15 ± 0.72	0.87 ± 0.59	0.015 *
Depression (CES-D), mean ± SD	1.94 ± 0.55	1.79 ± 0.46	0.105
Psychological wellbeing (PWB), mean ± SD			
Self-acceptance	4.65 ± 1.18	4.86 ± 1.00	0.377
Autonomy	3.94 ± 0.97	4.33 ± 1.00	0.024 *
Purpose in life	4.59 ± 1.18	4.69 ± 1.11	0.660
Positive relations with others	4.79 ± 1.11	4.71 ± 1.00	0.523
Personal growth	5.13 ± 0.95	5.02 ± 0.88	0.285
Environmental mastery	4.37 ± 1.09	4.48 ± 0.94	0.538
Emotional intelligence (TIEQUE), mean ± SD			
Assertiveness	4.62 ± 0.92	5.10 ± 1.16	0.011 *
Emotion regulation	4.95 ± 1.15	5.10 ± 1.23	0.461
Self-motivation	5.15 ± 1.10	5.03 ± 1.12	0.530
Emotion perception	4.91 ± 1.44	5.06 ± 1.39	0.557
Positive emotions (PSS), mean ± SD	3.72 ± 0.62	3.85 ± 0.52	0.192
Negative emotions (PSS), mean ± SD	2.60 ± 0.60	2.39 ± 0.59	0.044 *
Optimism (LOT-R), mean ± SD	3.68 ± 0.71	3.72 ± 0.63	0.733

* *p* value obtained with chi-squared test, Student´s t-test and Mann–Whitney U test. Monthly extra money: 5 categories, from nothing to more than USD 180; smoking and alcohol consumption frequency were measured, from 0 to 4 (never to more than 4 times in the week); sleep satisfaction (OVIEDO scale), from 1 to 7 (very unsatisfied to very satisfied); sleep quality (OVIEDO scale), from 1 to 5 (low quality to high quality); quality of food intake (Mini-Ecca scale), from 1 to 12 (very low quality to very high quality); frequency of food consumption outside the home and frequency of food consumption in excess, from 1 to 7 (less than once in a month to all the days); anxiety (GAD-7 scale), from 0 to 3 (never to almost all the days); depression (CES-D scale), from 1 to 4 (none of the days to all the days); subscales for psychological wellbeing (PWB), from 1 to 6 (totally disagree to totally agree); emotional intelligence (TEIQUE scale), from 1 to 7 (totally disagree to totally agree); positive and negative emotions (PSS scale), from 1 to 5 (never to almost always); and optimism (LOT-R), from 1 to 5 (totally disagree to totally agree).

**Table 2:** Descriptive data of the laboratory studied variables.

Variable	Women (*n* = 70)	Men (*n* = 64)	Laboratory Reference Values	Participants out of Range *n* (%)
8-OHdg, ng/mL	3.88 ± 0.98	3.73 ± 0.86	N.A.	N.A.
Leukocytes (103/μL), mean ± SD	6.82 ± 1.52	6.47 ±1.65	5.0–10.00	0 (0)
Lymphocytes	2.24 ± 0.50	2.10 ± 0.48	1.0–4.20	0 (0)
Monocytes	0.50 ± 0.14	0.52 ± 0.16	0.10–1.00	0 (0)
Neutrophils	3.87 ± 1.24	3.65 ± 1.38	1.50–7.00	W: 1 (1.4), M: 2 (3.1)
Eosinophils	0.14 ± 0.13	0.13 ± 0.08	0.05–0.40	W: 7 (10.0), M: 9 (14.1)
Basophils	0.04 ± 0.02	0.04 ± 0.02	0.01–0.05	W: 18 (25.7), M: 15 (23.4)
Hemoglobin (g/dL), mean ± SD	13.74 ± 1.27	16.29 ± 0.76	W: 12.00–16.00, M: 14.00–17.00	W: 7 (10.0), M: 13 (20.3)
Hematocrit (%)	42.13 ± 3.36	48.27 ± 2.31	W: 36.0–48.0, M: 36.0–52.0	W:8 (11.4), M: 3 (4.7)
Erythrocytes 10^6^/µL	4.71 ± 0.35	5.43 ± 0.33	W: 4.0–5.0, M: 4.5–6.2	W:15 (21.4), M: 0 (0.0)
Platelets (10^3^/μL), mean ± SD	282.79 ± 57.18	257.11 ± 52.82	141.00–400.00	W: 2 (2.9), M: 1 (1.6)
Glucose (g/dL), mean ± SD	88.59 ± 8.41	91.00 ± 15.81	74.00–106.00	W: 2 (2.9), M: 1 (1.6)
Urea (mg/dL), mean ± SD	25.92 ± 6.93	28.64 ± 7.49	16.6–48.5	W: 3 (4.3), M: 3 (4.7)
Blood urea nitrogen (BUN), mg/dL, mean ± SD	12.11 ± 3.23	13.38 ± 3.50	6.0–20.0	W: 4 (5.7), M: 4 (6.3)
Creatinine (mg/dL), mean ± SD	0.75 ± 0.12	0.94 ± 0.12	W: 0.50–0.90 M: 0.70–1.20	W: 0 (0.0), M: 3 (4.7)
Uric acid (mg/dL) mean ± SD	4.33 ± 1.02	6.03 ± 1.12	W: 2.4–5.7 M: 3.4–7.0	W: 0 (0.0), M: 12 (18.8)
Lipid levels (mg/dL), mean ± SD				
Total cholesterol	167.33 ± 29.39	172.65 ± 37.32	≤200.00	W: 12 (17.1), M: 16 (25.0)
High density lipoprotein (HDL)	51.93 ± 11.87	45.05 ± 10.37	W ≥ 45.00, M ≥ 35.00	W: 25 (37.5), M: 10 (15.6)
Low density lipoprotein (LDL)	96.29 ± 24.51	106.25 ± 31.02	≤100.00	W: 27 (38.6), M: 33 (51.6)
Triglycerides	94.75 ± 42.67	115.11 ± 82.01	≤150.00	W: 8 (11.4), M: 15 (23.4)
Total proteins (g/dL)	7.52 ± 0.40	7.68 ± 0.38	6.4–8.3	W: 3 (4.3), M: 3 (4.7)
Albumin (g/dL)	4.72 ± 0.23	5.04 ± 0.24	3.97–4.94	W: 10 (14.3), M: 42 (65.6)
Liver enzymes (U/L), mean ± SD				
AST	20.60 ± 18.18	31.71 ± 44.23	W ≤ 32.00, M ≤ 40.00	W: 4 (5.7), M: 6 (9.4)
ALT	17.99 ± 17.24	27.95 ± 20.18	W ≤ 33.00, M ≤ 41.00	W: 6 (8.6), M: 8 (12.5)
GGT	16.01 ± 6.83	23.17 ± 10.39	W ≤ 40.00, M ≤ 60.00	W: 1 (1.4), M: 0 (0.0)
ALP	78.49 ± 15.16	98.07 ± 27.75	W ≤ 104.00, M ≤ 129.00	W: 2 (2.9), M: 0 (0.0)
LDH	168.57 ± 34.82	191.08 ± 112.06	W ≤ 214.00, M ≤ 225.00	W: 4 (5.7), M: 5 (7.8)
Serum electrolytes				
Calcium, mg/dL	9.75 ± 0.30	9.97 ± 0.33	8.6–10-0	W: 8 (11.4), M: 22 (34.4)
Phosphorus, mg/dL	3.70 ± 0.48	3.63 ± 0.46	2.5–4.5	W: 3 (4.3), M: 1 (1.6)
Magnesium, mg/dL	2.05 ± 0.11	2.07 ± 0.14	W:1.7–2.2, M: 1.6–2.6	W: 2 (2.9), M: 0 (0.0)
Iron, μg/dL	84.33 ± 38.21	120.74 ± 40.72	33.0–193.0	W: 6 (8.6), M: 4 (6.3)
Sodium, meq/L	138.83 ± 1.90	139.41 ± 1.97	136.0–145.0	W: 3 (4.3), M: 2 (3.1)
Potassium, meq/L	4.52 ± 0.39	4.40 ± 0.37	3.5–5.10	W: 5 (7.1), M: 2 (3.1)
Chloride, meq/L	103.81 ± 1.89	102.71 ± 1.91	98.0–107.0	W: 3 (4.3), M: 1 (1.6)
Pancreatic enzymes				
Amilase, U/L	69.12 ± 25.69	66.71 ± 24.33	28.0–100.0	W: 8 (11.4), M: 6 (9.4)
Lipase, U/L	34.03 ± 12.05	31.63 ± 21.80	13.0–60.0	W: 2 (2.9), M: 1 (1.6)
Ultra-sensible C reactive protein (CRP), median (range)	0.95 (0.15–11.57)	0.83 (0.14–20.87)	0.1–3.0	W: 17 (24.3), M: 6 (9.4)
Urine exam test, median (range)				
Leukocyte esterase	0 (0–500)	0 (0–100)	0	W: 31 (44.3), M: 6 (9.4)
Erythrocytes in urine	1 (0–16)	0 (0–20)	0	W: 35 (50.0), M: 20 (31.3)
Leukocytes in urine	3.5 (1–30)	1 (1–6)	0	N.A.

8-OHdG: 8-deoxyguanosine, AST: aspartate aminotransferase, ALT: alanine aminotransferase, GGT: gamma glutamyl transferase, ALP: alkaline phosphatase, LDH: lactate dehydrogenase. W: women, M: men. N.A.: not applicable.

**Table 3:** Significant bivariate correlations between the studied variables and the levels of 8-deoxyguanosine (8OHdG).

Variables	Global Sample (*n* = 134)	Women (*n* = 70)	Men (*n* = 64)
Age	0.394 **	0.316 **	0.450 **
Schooling	0.375 **	0.298 *	0.434 **
With children	0.266 **	0.270 *	0.254 *
With job	0.181 *	0.215	0.125
Sum of diseases in the last 6 months	−0.030	0.175	−0.272 *
Antioxidants/vitamins supplements (Yes = 1, No = 0)	−0.137	−0.315 **	0.089
Frequency of vitamins/antioxidants Supplements consumption	−0.159	−0.378 *	0.100
Cholesterol	0.230 *	0.135	0.224
Low density lipoprotein (LDL)	0.246 **	0.293 *	0.206
Glucose	−0.111	−0.245 *	0.074
Phosphorus	−0.256 **	−0.335 *	−0.239
Potassium	0.182 *	0.132	0.212
Erythrocytes in urine	0.175 *	0.076	0.381 **
Highly sensitive C reactive protein (hs-CRP) ^†^	−0.003	0.018	−0.046

* *p* < 0.05, ** *p* < 0.01. Correlations performed with the Pearson and Spearman correlation tests. ^†^ This variable was not significant for any sample; however, we included it here to better visualize these results.

**Table 4:** Multivariate regression analysis for 8-OHdG in the global sample.

Variable	B	Beta Coefficient	Significance	Change in R^2^	Tolerance
Constant	1.920	-	0.010	-	-
Age	0.037	0.390	0.000	0.137	0.861
Schooling	0.396	0.301	0.000	0.060	0.909
Glucose	−0.015	−0.206	0.008	0.048	0.896
Erythrocytes in urine	0.078	0.249	0.001	0.035	0.946
Frequency of antioxidants consumption	0.123	−0.173	0.019	0.031	0.978
Depression	0.366	0.204	0.008	0.030	0.911
Amylase	0.006	0.151	0.047	0.021	0.916

R of the model: 0.601.

**Table 5:** Multivariate regression analysis for 8-OHdG in the women’s sample.

Variable	B	Beta Coefficient	Significance	Change in R^2^	Tolerance
Constant	6.016	-	0.000	-	-
Phosphorus	−0.635	−0.311	0.001	0.120	0.951
Frequency of antioxidants consumption	−0.271	−0.353	0.000	0.096	0.901
Erythrocytes in urine	0.092	0.297	0.002	0.082	0.903
LDL-cholesterol	0.011	0.284	0.002	0.049	0.934
Glucose	−0.030	−0.236	0.010	0.049	0.951
Sum of diseases	0.132	0.236	0.012	0.056	0.911
Lymphocytes	0.485	0.247	0.007	0.047	0.967
Eosinophils	−1.430	−0.185	0.039	0.035	0.958
LDH	0.005	0.181	0.048	0.031	0.935

R of the model: 0.751.

**Table 6:** Multivariate regression analysis for 8-OHdG in the men’s sample.

Variable	B	Beta Coefficient	Significance	Change in R^2^	Tolerance
Constant	1.188	-	0.407	-	-
Erythrocytes in urine	0.066	0.196	0.014	0.230	0.956
Glucose	−0.016	−0.292	0.002	0.105	0.707
Schooling	0.524	0.450	0.000	0.061	0.718
Age	0.045	0.485	0.000	0.084	0.703
Potassium	0.736	0.315	0.000	0.068	0.887
Erythrocytes	−0.662	−0.256	0.003	0.038	0.851
Uric acid	0.181	0.236	0.006	0.033	0.857
Lipase	−0.008	−0.204	0.018	0.028	0.810
Monthly extra money	0.122	0.185	0.032	0.026	0.809
Leucocytes in urine	0.141	0.162	0.048	0.023	0.884

R of the model: 0.835.

## Data Availability

Data are contained within the article and supplementary materials.

## Supplementary Materials

The following supporting information can be downloaded at: https://www.mdpi.com/article/10.3390/antiox13010110/s1.
