# Supplementary material for: Association between Oxidative Stress with Psychological and Biochemical Variables in a Sample of Healthy Mexican People: A Cross-Sectional Study
Source: Antioxidants (Basel). 2024 Jan 17;13(1):110. doi: 10.3390/antiox13010110 (PMC10812661; doi:10.3390/antiox13010110)
Supplement: Supplementary file 1 [file antioxidants-13-00110-s001.zip › antioxidants-2778265-supplementary.pdf]

Items included in the emotional intelligence subscales of the TEIQUE scale

Instruction: Please indicate the degree of agreement or disagreement with the following statements. Answer options: 1: absolutely disagree- 7: absolutely agree

Self-motivation

1.- On the whole, I'm a highly motivated person (En general soy una persona con alta motivación)

2.- Sometimes, it feels like I'm producing a lot of good work effortlessly (Me cuesta trabajo esforzarme ante una tarea)

3.- I normally find it difficult to keep myself motivated (Me cuesta motivarme por lo que hago)

4.- I tend to get a lot of pleasure just from doing something well (Tiendo a obtener una gran satisfacción simplemente con hacer algo bien)

5.- I lose interest in what I do quite easily (Suelo perder interés por lo que hago)

Emotion regulation

1.- I tend to get "carried away" easily (Tiendo a enfadarme o exaltarme con facilidad)

2.- I usually find it difficult to regulate my emotions (Me cuesta controlar mis emociones)

3.- I´m usually able to calm down quickly after I´ve got mad at someone (Suelo calmarme con rapidez después de haberme enfadado con alguien)

4.- I´m usually able to find ways to control my emotions when I want to (Puedo encontrar diferentes maneras de controlar mis emociones cuando lo deseo)

5.- I know how to snap out my negative moods (Sé cómo animarme cuando me siento mal)

6.- When someone offends me, I´m usually able to remain calm (Cuando alguien me ofende soy capaz de mantener la calma)

Emotion perception

1.- I often find it difficult to recognize what emotion I am feeling (Me cuesta reconocer mis propias emociones)

2.- I´m never really sure what I´m feeling (Nunca estoy realmente seguro/a de lo que estoy sintiendo)

3.- Many times, I can´t figure out what emotion I´m feeling (Muchas veces no consigo tener claro qué emoción estoy sintiendo)

4.- On the whole, I find it difficult to describe my feelings (En general me resulta difícil describir mis sentimientos)

5.- Most of the time, I know exactly why I feel the way I do (La mayoría de las veces sé exactamente por qué siento lo que estoy sintiendo en ese momento)

Assertiveness

1.- When I disagree with someone, I usually find it easy to say so (Cuando estoy en desacuerdo con alguien me resulta fácil decirlo)

2.- I tend to “back down” even if I know I´m right (En una discusión tiendo a ceder incluso cuando sé que estoy en lo cierto)

3.- I usually find it difficult to express myself clearly (Me resulta difícil expresarme con claridad)

4.- I would normally defend my opinions even if it meant arguing with important people (Normalmente defiendo mis opiniones incluso si tengo que discutir con gente importante)

5.- I tend to speak well and clearly (Tiendo a hablar bien y claramente)

6.- I often find it difficult to stand up for my rights (En muchas ocasiones me resulta difícil defender mis derechos)
